# Pulmonary Endarterectomy for Chronic Thromboembolic Pulmonary Hypertension: A Systematic Review of the Most Updated Literature

**DOI:** 10.3390/jcm11236976

**Published:** 2022-11-26

**Authors:** Eleonora Faccioli, Vincenzo Verzeletti, Martina Perazzolo Marra, Annalisa Boscolo, Marco Schiavon, Paolo Navalesi, Federico Rea, Andrea Dell’Amore

**Affiliations:** 1 Thoracic Surgery Unit, University Hospital of Padua, 35128 Padua, Italy; 2 Cardiology Unit, University Hospital of Padua, 35128 Padua, Italy; 3 Intensive Care Unit, University Hospital of Padua, 35128 Padua, Italy

**Keywords:** pulmonary hypertension, pulmonary endarterectomy, chronic thromboembolic pulmonary hypertension, outcomes

## Abstract

Pulmonary endarterectomy (PEA) is the treatment of choice in case of chronic thromboembolic pulmonary hypertension (CTEPH). PEA is performed by an increasing number of surgeons; however, the reported outcomes are limited to a few registries or to individual centers’ experiences. This systematic review focuses on pre-operative evaluation, intra-operative procedure and post-operative results in patients submitted to PEA for CTEPH. The literature included was searched using a formal strategy, combining the terms “pulmonary endarterectomy” AND “chronic pulmonary hypertension” and focusing on studies published in the last 5 years (2017–2022) to give a comprehensive overview on the most updated literature. The selection of the adequate surgical candidate is a crucial point, and the decision should always be performed by expert multidisciplinary teams composed of surgeons, pulmonologists and radiologists. In all the included studies, the surgical procedure was performed through a median sternotomy with intermittent deep hypothermic circulatory arrest under cardiopulmonary bypass. In case of residual pulmonary hypertension, alternative combined treatments should be considered (balloon angioplasty and/or medical therapy until lung transplantation in highly selected cases). Short- and long-term outcomes, although not homogenous across the different studies, are acceptable in highly experienced CTEPH centers.

## 1. Introduction

Chronic thromboembolic pulmonary hypertension (CTEPH) is a rare condition characterized by symptomatic pulmonary hypertension (mean > 25 mmHg) with a pulmonary capillary wedge pressure of ≥15 mmHg and persistent lung perfusion defects (at least one segmental) despite prolonged anticoagulation therapy (3 to 6 months) [1], with a reported annual incidence of 3–5 cases/100,000 population [1,2]. 

The diagnosis of this condition is often insidious, as the main symptoms (fatigue, dyspnea, exercise intolerance, and syncope) are not specific, with a consequent significant diagnostic delay [3]. 

According to the European Respiratory Society/European Society of Cardiology (ERS/ESC) guidelines [4], pulmonary endarterectomy (PEA) is considered the treatment of choice in symptomatic patients with surgically accessible thromboembolic lesions. One of the major concerns of this complex surgical procedure, performed through median sternotomy with extracorporeal circulation and intermittent circulatory arrest in deep hypothermia (HCA), is the correct selection of the candidate. In general, the patient’s symptoms and comorbidities, the severity of pulmonary hypertension and right heart dysfunction, and risk–benefit analysis are the most important factors of the decision-making process [5,6].

Technical aspects, especially the location and the type of thromboembolic lesions, are also considered as selection criteria. The Jamieson classification [7] is historically the most commonly utilized in clinical practice; it defines the disease as Type 1 (fresh thrombus in the main lobar arteries), Type 2 (organized thrombus and intimal thickening proximal to segmental arteries), Type 3 (intimal thickening fibrosis in distal segmental arteries, with the surgical plane raised at each segmental level) or Type 4 (distal arteriolar vasculopathy with removal of the normal intimal layer and no intraluminal disease). Recently, a new intra-operative classification, proposed by the San Diego Group, categorizes thromboembolic lesions into levels based on their location (Table 1). As a general rule, the lesions located proximally (main, lobar and segmental arterial branches) are those amenable for PEA, but experienced surgeons do not arbitrarily exclude patients based only on the location of the disease; in fact, this procedure can successfully be performed in cases of more distal lesions with good functional results [8,9]. In most recent times, experienced surgeons have proposed that PEA should be considered in all patients who have evidence of thrombotic disease, including those with more distal lesions [6,8,9,10]. 

Always with regard to technical aspects, alternative techniques to HCA have been developed over the years, such as selective cerebral perfusion or models of left and right heart chamber venting. Even though PEA on HCA remains the technique of choice, these alternatives were found to be effective in preventing bronchial artery back bleeding and avoiding side effects of HCA, such as metabolic changes induced by hypothermia and postoperative neurological complications [11,12,13,14].

Outcomes of PEA depend on several factors such as the chronicity and distribution of disease, pre-operative pulmonary vascular resistance (PVR), New York Heart Association (NYHA) functional class, comorbidities and, in particular, the CTEPH team’s experience [8,9,10,15]. It is widely reported that peri-operative mortality rates decline with increasing surgical expertise [16], having in mind that experienced surgeons in PEA are defined as those who performed >20 PEAs in the year they started to assess study cases, and/or >30 in the year before they started to assess study cases and/or 40 cases in the 3 years before they started to assess study cases [15].

The two most important complications which can affect post-operative outcomes are residual pulmonary hypertension and reperfusion pulmonary edema. Both situations, in the immediate peri-operative period, can be successfully managed via extracorporeal membrane oxygenation (ECMO), which can improve the function of the right ventricle and, subsequently, gas exchange and cardiac output. For this reason, as recommended by the CTEPH task force, PEA centers should also have expertise in ECMO management to deal with potential intra- and/or post-operative complications [17]. 

In case of persistent post-operative pulmonary hypertension (PH), in the absence of a specific cut-off, definitions and guidelines on the optimal strategy, complementary treatments such as balloon pulmonary angioplasty (BPA) or medical treatments (for example, Riociguat) can be considered in addition to the surgical procedure [18,19,20]. 

In this context, lung transplantation for CTEPH can be considered as a salvage option only in highly selected cases and in case of failure or exclusion from the above-mentioned options in case of residual PH or in case of inability to wean from ECMO after PEA [21]. Unfortunately, reports on lung transplantation in CTEPH are anecdotal and outcomes can only be extracted from few reports and are not satisfactory.

The purpose of this study is to provide a systematic review of the current literature on outcomes of patients submitted to PEA for CTEPH, with particular attention to post-operative short- and long-term outcomes. 

## 2. Materials and Methods

### 2.1. Literature Search Strategy

A systematic review of the literature was conducted using a formal strategy (https://www.ncbi.nlm.nih.gov/pubmed; accessed on 15 September 2022) and according to the Preferred Reporting Items for Systematic Reviews and Meta-Analysis (PRISMA) Statement [22]. To retrieve all the publications dealing with the topic of interest, the query string was composed as “Pulmonary Endarterectomy AND Chronic pulmonary hypertension”. 

Subsequently, the authors independently assessed the eligibility of the articles by screening titles and abstracts, then decided on their inclusion after reading the full texts of the selected papers.

### 2.2. Inclusion and Exclusion Criteria

After the search using the keywords mentioned above, a total of 1248 studies were identified. In this systematic review, eligible studies were those focused on surgically treated patients, concerning pre-operative assessment, intra-operative course and post-operative outcomes. All the selected publications were limited to human subjects and in English language. We focused our research only on studies published in the last five years (2017–2022) in order to limit our review to the most updated and modern series.

Abstracts, case series, reviews, meta-analyses, editorials, how-to guides and expert opinions were excluded, as were studies with less than 40 patients. 

Two investigators (E.F, V.V) independently reviewed each article. Discrepancies between the two reviewers were resolved by discussion and consensus.

The main outcomes considered in this review article were the need for post-operative extracorporeal membrane oxygenation (ECMO) support, intensive care unit (ICU) and in-hospital length of stay, in-hospital mortality, residual pulmonary hypertension after PEA and overall survival. Fourteen studies met the inclusion criteria and were considered for this review. The majority of these studies were heterogeneous, reporting different kinds of outcomes. This made the analysis difficult, so we paid attention to not overinterpreting any of these results, only presenting them with the given limitations.

**Figure 1:**
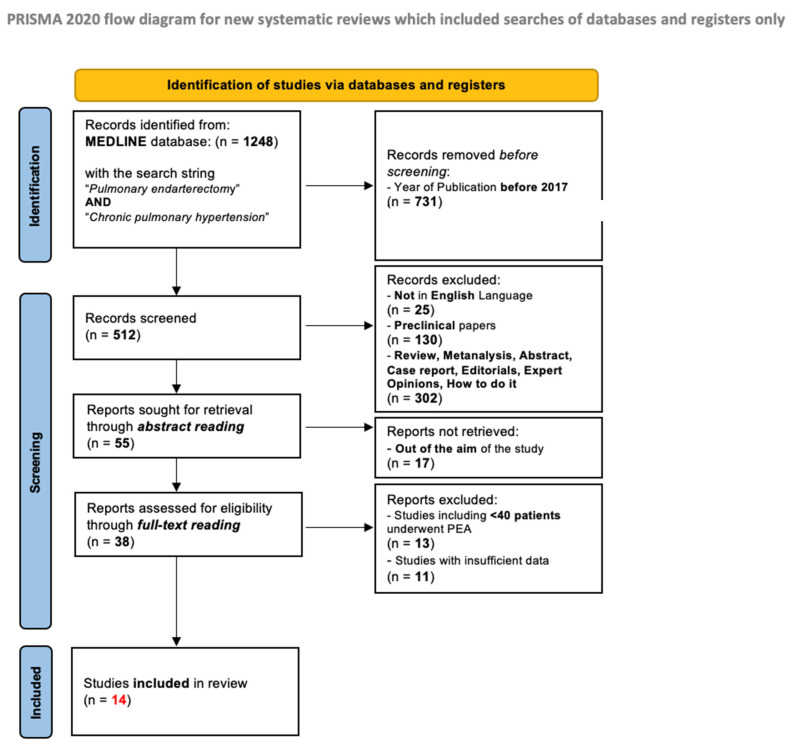
PRISMA flow diagram for this systematic review [22].

The PRISMA flow diagram with the search strategy is presented in Figure 1.

## 3. Results

### 3.1. Baseline Characteristics 

The number of patients submitted to PEA in the different studies was extremely heterogenous, ranging from 44 [23] to 1324 [24], with a median age from 51 [25,26] to 61 [24] years old. Considering the entire population of these studies (4074 patients), 1757 (43.1%) were female, with a median age of 57 years old (IQR_25–75_ 56–59). All the baseline data are summarized in Table 2.

### 3.2. Pre- and Intra-Operative Data

Pre- and intra-operative data are reported in Table 2. The pre-operative right heart catheterization showed pre-operative PVR values ranging from 552 dyn·s·cm^−5^ [27] to 1536.6 dyn·s·cm^−5^ [28], with a median calculated value of 639 dyn·s·cm^−5^ (IQR_25–75_ 626–778), while the mean pulmonary artery pressure (mPAP) ranged from 42 mmHg [26] to 49.7 mmHg [25], with a median, among all the studies, of 46 mmHg (IQR_25–75_ 44–47.5). 

Seven studies reported data about patients treated with medications for PH before surgery. The rate of treated patients ranged from 19.3% [29] to 56.8% [23]. These seven studies included a total of 3024 patients, of which 746 (31.9%) were pre-operatively treated with PH-targeted therapy. The time of HCA, reported by ten studies, was between 28 min [30] and 66 min [26], with a median calculated value of 38.5 min (IQR_25–75_ 37–46.5). The cardiopulmonary bypass time was reported in nine studies and ranged from 227.3 [28] to 359.9 min [26], with a calculated median time of 234.5 min (IQR_25–75_ 227.75–245.75) across the different studies.

### 3.3. Post-Operative Data and Short- and Long-Term Outcomes

Five studies reported data about the need for post-operative extracorporeal membrane oxygenation (ECMO) support, with rates ranging from 3.5% [31] to 9% [28].

The lengths of intensive care unit (ICU) and in-hospital stays were reported by eight and six studies, respectively, with values ranging from 4 [24,29] to 10 [30] days and from 12.6 [32] to 45 [33] days, respectively.

The in-hospital mortality rate was reported in nine studies, with rates from 1.7% [31] to 8.4% [29,34]. Regarding post-operative mPAP and PVR, their values ranged from 18 [23] to 33.4 mmHg [29] with a median value of 22 mmHg (IQR_25–75_ 21–24.5) and from 206 [31] to 462.3 dyn·s·cm^−5^ [30] with a median value of 219 dyn·s·cm^−5^ (IQR_25–75_ 208–264), respectively. 

Residual PH rates after PEA ranged from 8.2% [32] to 41.9% [34], and they were reported in seven studies. The overall survival rate, reported in five studies, ranged from ~82.5% [29] to 98% [35] at 1 year and from 77% [29] to 89.2% [33] at 5 years. Data on short- and long-term outcomes are reported in Table 3.

**Table 2:** Baseline, pre-operative, and intra-operative features of patients treated with PEA.

#	Author, Year	No. of Patients	Sex	Age (Years)	BMI (Kg/m^2^)	Pre-PEA PVR (dyn·s·cm^−5^)	Pre-PEA mPAP (mmHg)	Pre-Operative PH Therapy	Time of Hypothermic Circulatory Arrest (min)	CPB Time (min)
1	Korsholm et al., (2017) [29]	239	M 54.8% F 45.2%	60	NR	857	48.4	19.3%	38	NR
2	Sihag et al., (2017) [32]	134	M 60.4% F 39.6%	54	NR	639	53	NR	37	180
3	Amsallem et al., (2018) [28]	486	M 51.7% F 48.3%	60.7	26.4	1536.6	45.4	NR	30.7	227.3
4	Yanaka et al., (2018) [23]	44	M 36.4% F 63.6%	58.5	22	950.4	45.1	56.8%	NR	NR
5	Quadery et al., (2018) [35]	272	M 55% F 45%	58	30	616	47	27%	NR	NR
6	Sakurai et al., (2019) [33]	122	M 72% F 28%	56	NR	832	47	NR	138	248
7	Zhang et al., (2019) [25]	123	M 66.6% F 34.4%	51.5	23	636	49.7	NR	39	239
8	Yan et al., (2019) [31]	58	M 63.8% F 36.2%	48	NR	724	49	NR	40	230
9	Newnham et al., (2020) [24]	1324	M 53.2% F 46.8%	61	NR	668.8	45	29%	37	321
10	Nagel et al., (2020) [36]	45	M 51.1% F 48.9%	59	25.2	648.8	44	NR	NR	NR
11	Delcaux et al., (2020) [30]	80	M 37% F 63%	57	27.9	892.8	45.9	55%	28	227
12	Kerr et al., (2021) [27]	566	M 52.7% F 47.3%	57	30.4	552	44	39.4%	NR	NR
13	Miyahara et al., (2021) [34]	499	M 54.9% F 45.1%	57.5	NR	813.7	44.8	34.9%	31.4	127.5
14	Liu et al., (2022) [26]	82	M 64.6% F 35.4%	50.8	24.3	1143.1	42	NR	66	359.9

BMI: body mass index; PEA: pulmonary endarterectomy; PVR: pulmonary vascular resistance; mPAP: mean pulmonary artery pressure; PH: pulmonary hypertension; CPB: cardiopulmonary bypass; M: male; F: female; NR: not reported.

**Table 3:** Short- and long-term outcomes of patients treated with PEA.

#	Author, Year	Postoperative ECMO Support	ICU Stay (Days)	In-Hospital Stay (Days)	In-Hospital Mortality	Post PEA PVR (dyn·s·cm^−5^)	Post PEA mPAP (mmHg)	Residual PH after PEA	OS
1	Korsholm et al., (2017) [29]	3.8%	4	NR	8.4%	350	33.4	17.5%	1~82.5% 3 84% 5 77%
2	Sihag et al., (2017) [32]	NR	4.6	12.6	3.7%	NR	22	8.2%	NR
3	Amsallem et al., (2018) [28]	9%	NR	20.2	NR	NR	NR	NR	NR
4	Yanaka et al., (2018) [23]	NR	NR	NR	NR	340.6	18	30.7%	NR
5	Quadery et al., (2018) [35]	NR	NR	NR	NR	NR	NR	40%	1~98% 3~90% 5~83%
6	Sakurai et al., (2019) [33]	25%	7	45	7%	219	20	NR	1 91.8% 3 89.2% 5 89.2%
7	Zhang et al., (2019) [25]	NR	5.5	15.2	NR	NR	NR	NR	NR
8	Yan et al., (2019) [31]	3.5%	6	NR	1.7%	206	22	12%	NR
9	Newnham et al., (2020) [24]	5.5 %	4	14.5	3.7%	254.4	25	35.01%	NR
10	Nagel et al., (2020) [36]	NR	NR	NR	NR	264	30	24%	NR
11	Rezaiguia- Delclaux et al., (2020) [30]	NR	10	NR	2.5%	462.3	25.7	NR	NR
12	Kerr et al., (2021) [27]	NR	NR	NR	3.9%	208	24	NR	1 94.4% 3 NR 5 NR
13	Myahara et al., (2021) [34]	NR	NR	NR	8.4%	259.7	23.5	41.9%	1~89% 3~88% 5 84.8%
14	Liu et al., (2022) [26]	NR	6.9	19.7	6.1%	396.8	19.8	29.3%	NR

ECMO: extracorporeal membrane oxygenation; ICU: intensive care unit; PEA: pulmonary endarterectomy; PVR: pulmonary vascular resistance; mPAP: mean pulmonary artery pressure; PH: pulmonary hypertension; OS: overall survival; NR: not reported.

## 4. Discussion

The present systematic review includes fourteen recent studies reporting pre-, intra- and post-operative data on patients submitted to PEA for CTEPH. The analysis was made difficult by the heterogeneity of these studies, concerning both the patients’ number and the outcomes reported, so we attempted not to overinterpret the results. 

The first important consideration concerns the reported values of pre-PEA PVR, which ranged between 552 dyn·s·cm^−5^ [27] and 1536.6 dyn·s·cm^−5^ [28]. Different papers have already shown that high pre-operative PVR values increase mortality [3,37,38]; in particular, it has been demonstrated that in-hospital mortality is three times higher in patients with pre-operative PVR > 1200 dyn·s·cm^−5^ [3]. Among all the collected studies, Amsallem et al. [28] and Liu et al. [26] reported the highest pre-operative PVR; the latter study also reported an in-hospital mortality rate of 6.1%, which was lower than those reported by Korsholm et al. [29], Sakurai et al. [33] and Myahara et al. [34]. Despite pre-operative PVR needing to be taken into consideration in the selection process of adequate candidates, it has been established that the pre-operative PVR value should not be considered alone as an exclusion criterion for PEA [39], as high pre-operative PVR is not necessarily related to worse post-operative outcomes.

The second consideration concerns the need for pre-operative therapy in patients affected by CTEPH and candidates for surgery. The reported data are extremely heterogeneous, with rates ranging from 19.3% [29] to 56.8% [23]. The utilization of pulmonary arterial hypertension (PAH)-targeted therapy remains controversial in patients with CTEPH who are not suitable for PEA, but the changes in pulmonary vessels and hemodynamics observed in CTEPH are similar to those in patients affected by PAH from the histopathological point of view as well [40]. These findings suggest that PAH and CTEPH probably share common pathophysiological contexts, providing reasonable evidence to suggest that patients with CTEPH can take PAH-targeted drugs. Targeted therapies include soluble guanylate cyclase stimulator (sGC), endothelin receptor antagonists (ERAs), phosphodiesterase-5 inhibitors (PDE5i), prostacyclin and its analogs, and prostacyclin receptor agonists. The rationale for the use of medical treatments before surgery is to optimize hemodynamics, potentially improving morbidity and mortality associated with the surgical procedure, but the use of targeted therapies in case of CTEPH before PEA is more controversial. Some authors have shown that the use of PAH-targeted therapies could be associated with a significant delay in referral for surgery, with no benefit in the outcomes [41]; more recently, similar results were reported in a prospective international registry [15] reinforcing the lack of evidence for using PAH-targeted therapy before PEA. On the other hand, the use of medical therapies before PEA in patients with CTEPH with severe hemodynamic impairment was associated with better outcomes in more severe patients with low cardiac output [34].

Concerning short-term outcomes, in-hospital mortality rates among the different studies ranged between 1.7% [31] and 8.4% [29,34]; Korsholm et al. [29], Sakurai et al. [33] and Myahara et al. [34] reported the highest in-hospital mortality, but this did not seem to be correlated to a worse pre-operative condition of the patients.

Concerning long-term outcomes, the rate of residual PH after surgery ranged between 8.2% [32] and 41.9% [34]. Residual or persistent PH after CTEPH is a common finding, affecting 25–30% of patients after PEA [42,43]. To date, there is no consensus on the definition of persistent residual PH after PEA, with no established threshold values [44]; in addition, the measurement of residual/persistent PH is made at different moments after surgery (immediately or after several months in different studies), and post-operative right heart catheterization is not routinely performed. The correlation between residual PH and short- and long-term post-operative outcomes is also a matter of debate: a prospective study from the Cambridge group showed that the 5-year survival rate did not differ between patients with a post-operative mPAP of <30 mmHg and those with values of ≥30 mmHg [45]. Recently, some authors found a strong and clinically relevant association between residual PH and long-term survival after PEA [46]. Again, the recent ERS statement on CTEPH has acknowledged that significant residual PH is a challenge to treat early post-operatively and is the most common cause of in-hospital mortality [44]. Patients with residual PH after PEA should be submitted to other treatments after surgery, such as balloon pulmonary angioplasty (BPA). BPA is an effective treatment after PEA, but it could be more technically challenging as a treatment of residual PH after CTEPH than for inoperable CTEPH because of post-operative vascular changes, such as fibrotic occlusion or aneurismal dilatation, with a higher risk of vascular injuries or complications. Severe hemoptysis requiring embolization has been described in case of BPA after PEA [47]. Another option in case of residual PH is Riociguat, recently approved by several clinical trials [19,20,48]. Recently, pulmonary artery denervation (PADN) has also been described as feasible for patients with residual PH. The fluoroscopy-guided PADN procedure erogates fractionated radiofrequency energy point by point in the pulmonary artery [49]. A recent randomized trial compared patients treated via PADN with those receiving Riociguat for residual pulmonary hypertension, showing better outcomes in the PADN group with a significant reduction in PVR and improvement in the 6-min walking test [50]. The treatment of CTEPH is increasingly moving towards multimodality to obtain improvements in long-term outcomes; for this reason, it is advisable that surgery should be supplemented by other treatments (such as BPA, medical therapies and PADN), especially in the most challenging cases.

In conclusion, it is our assessment that the best treatment for CTEPH should be selected by balancing therapeutic efficacy with expected adverse events. Although the reported outcomes are not homogeneous, and there could be some interpretation bias mainly due to the retrospective nature of these studies, different patient populations, surgical techniques and centers’ expertise levels, PEA is feasible, with acceptable reported outcomes in highly experienced centers [51]. Surgery for CTEPH should be always considered as a part of comprehensive treatment according to lesion location, comorbidities and residual PH.

## Figures and Tables

**Table 1:** New proposed UCSD classification.

Levels	Disease Type
Level 0 (old Type 4)	No evidence of CTEPH
Level I (old Type 1 or 2)	Disease in main PA
Level IC (new)	Complete occlusion of one lung
Level II (old Type 1 or 2)	Disease starting in lobar arteries
Level III (old Type 3)	Disease starting in segmental arteries
Level IV (old Type 3)	Disease starting in sub-segmental arteries

PA: pulmonary artery; CTEPH: chronic thromboembolic pulmonary hypertension.

## Data Availability

All the data are available in the respective studies in the MEDLINE database.
